# Serious takotsubo cardiomyopathy: an autopsy case presenting severe irreversible myocardial damage after frequent episodes of recurrence

**DOI:** 10.1186/s13000-020-01006-x

**Published:** 2020-07-21

**Authors:** Kenichi Mizutani, Akihiro Shioya, Yasuyo Hirose, Ryuhei Saito, Sohsuke Yamada

**Affiliations:** 1 Depertment of Pathology and Laboratory Medicine, Kanazawa Medical University, 1-1 Daigaku, Uchinada, Kahoku, Ishikawa 920-0293 Japan; 2 Kanazawa Medical University, 1-1 Daigaku, Uchinada, Kahoku, Ishikawa 920-0293 Japan; 3 Depertment of Cardiology, Kanazawa Medical University, 1-1 Daigaku, Uchinada, Kahoku, Ishikawa 920-0293 Japan

**Keywords:** Takotsubo cardiomyopathy, Case report, Serious, Irreversible, Recurrence

## Abstract

**Background:**

Takotsubo cardiomyopathy is characterized by transient dysfunction of the medial to apical segment of the left ventricle. Recurrence within a few months or years has been reported and serious complications, including arrhythmia, acute cardiac shock and cardiac rupture, can arise; however, recurrence is rare and takotsubo cardiomyopathy is typically a reversible functional disorder.

**Case presentation:**

A 91-year-old Japanese woman with a past medical history of angina pectoris, hypertension and uterine carcinoma noted bilateral axillary pain and presented herself to an emergency room. Although the pain improved and she went home, there were several subsequent episodes of recurrent chest pain. At approximately 1 week after the onset, she was hospitalized as her symptom worsened. Electrocardiography showed low voltage in limb and chest leads, and ST-segment elevation in leads II, III, aVF and V3 to V6. Echocardiography revealed medial to apical dyskinesia and basal hypercontractility of the left ventricle, and cardiac tamponade. Pericardiocentesis improved the symptom, but not her cardiac dysfunction. At 3 days after her admission, cardiopulmonary resuscitation was performed due to ventricular fibrillation. She died on the 5th day of admission (2 weeks after the onset). At autopsy, the left ventricle was dilatated and the apical ventricular wall was thin. On microscopy, remarkable wavy change and thinning of myocardium were diffusely observed, especially at the apex and the anterior to lateral wall of the left ventricle, interventricular septum and right ventricle, intermingled with interstitial fibrosis, hemorrhage and neutrophil infiltration. Contraction band necrosis was mainly observed on the posterior to inferior wall of the left ventricle.

**Conclusion:**

Our case showed severe morphological myocardial change after several chest pain episodes that were considered to be takotsubo cardiomyopathy. This notable case suggests that the frequent recurrence of serious takotsubo cardiomyopathy is life threatening and can lead to irreversible serious myocardial degeneration.

## Background

Takotsubo cardiomyopathy, which was first described in Japan in 1990 [1], is characterized by transient dysfunction of the medial to apical segment of the left ventricle. The word takotsubo relates to a special shaped pot (octopus pot) and is used to describe the characteristic ballooning of the left ventricular apex [1]. It mostly affects elderly women and is often preceded by emotional or physical event; however, the cause of takotsubo cardiomyopathy has not been fully elucidated [1]. Takotsubo cardiomyopathy is often clinically diagnosed and the Mayo Clinic diagnostic criteria are widely used for this purpose [2, 3]. According to these diagnostic criteria, obstructive coronary disease, pheochromocytoma and myocarditis should be excluded. New electrocardiographic abnormalities, such as ST-segment elevation or elevation in cardiac troponin are observed. Reported recurrence rates range from 0 to 15% [4–8]. Although a few papers reported that the earliest time of recurrence was 8 days [4, 6], in most cases, a relapse occurs within a few months or years [1, 4, 6, 9–13]. Severe complications, including arrythmia, acute cardiac shock and cardiac rupture may arise [1, 4, 14–18]; however, takotsubo cardiomyopathy is typically a reversible functional disorder and autopsy cases are rare [19]. In most cases, macroscopic study of the heart shows focal damage, such as rupture, or the absence of structural change [20, 21]. Histologically, contraction band necrosis of the myocardium is commonly recognized [14, 15, 20–22], and a few papers have described thinning of the myocardium or interstitial fibrosis of the heart [17, 19, 20, 22].

We report a notable autopsy case of takotsubo cardiomyopathy with several episodes of recurrence within 2 weeks before death, which broadly presented severe irreversible myocardial damage.

## Case presentation

**Fig. 1:**
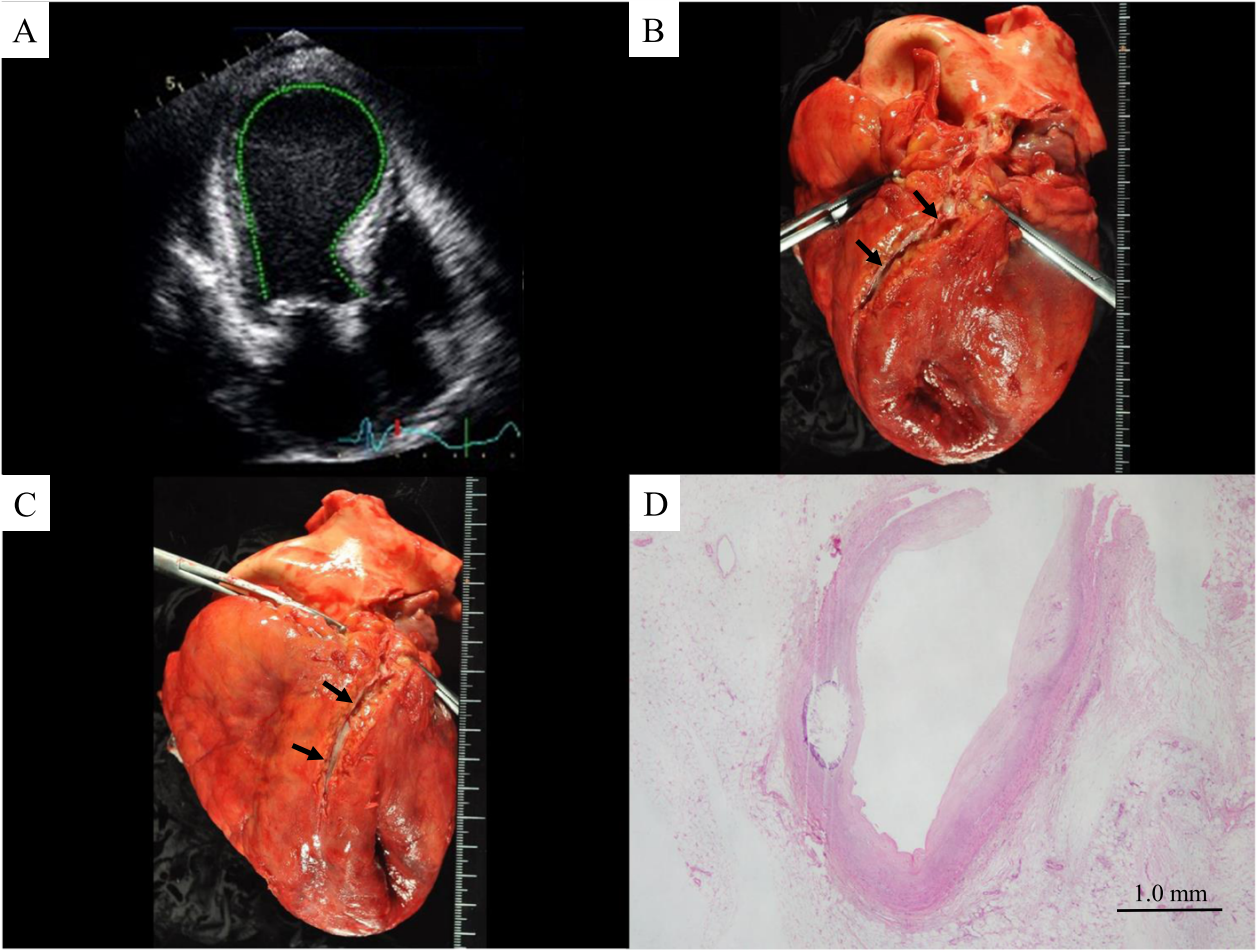
Echocardiography (**a**) and a macroscopic view (**b**, **c**) of the heart, and cross section of the coronary artery (**d**). **a** Echocardiography showed medial to apical dyskinesia and hypercontractility of the basal segments of the left ventricle, which seemed to have a takotsubo-like appearance. **b**, **c** The heart showed a takotsubo-like shape. The epicardium had a reddish color and rough surface. There were no findings of thrombus, embolism or obstruction in the coronary arteries (arrow). **d** Although there were mild intimal thickening and calcification of the coronary arteries, there was not severe stenosis of them. Bar = 1.0 mm (H&E staining; original magnification: × 20)

A 91-year-old Japanese woman with a past medical history of angina pectoris, hypertension and uterine carcinoma noted bilateral axillary pain and presented herself to an emergency room. As a physical examination and chest roentgenography showed no emergent findings and her pain improved, she returned home. However, she subsequently experienced several episodes of recurrent chest pain. At approximately 1 week after the onset, she was hospitalized due to continuous dyspnea and left chest pain. On examination, the patient was alert. Her body temperature was 36.4 °C, her pulse was 110 beats per minute, her blood pressure was 147/98 mmHg, and her respiratory rate was 28 breaths per minute. Her oxygen saturation was 98% on oxygen (6 L/min). A blood test revealed high levels of brain natriuretic peptide (BNP; 3431.5 pg/mL), creatine kinase (CK; 303 U/L), CK-MB (31 U/L), troponin T (0.813 ng/mL), C-reactive protein (CRP; 7.21 mg/dL), potassium (5.2 mEq/L), blood urea nitrogen (BUN; 41 mg/dL), creatinine (2.04 mg/dL), aspartate aminotransferase (AST; 68 U/L), and alanine aminotransferase (ALT; 35 U/L). Her red blood cell count was slightly low (3.61 × 10^6^/μL). Her white blood cell count, platelet count, and sodium and chlorine levels were within the normal ranges. Electrocardiography revealed sinus rhythm, low voltage in limb and chest leads, and ST-segment elevation in leads II, III, aVF and V3 to V6. Echocardiography revealed medial to apical dyskinesia and basal hypercontractility of the left ventricle, which seemed to have a takotsubo-like shape (Fig. 1a), and cardiac tamponade. After the drainage of 400 mL of hemorrhagic pericardial effusion by pericardiocentesis, the patient’s symptoms improved; however, the cardiac dysfunction did not. Coronary angiography was not performed due to her age and low kidney function. At 3 days after her admission, cardiopulmonary resuscitation was performed for loss of consciousness due to ventricular fibrillation. The patient’s blood pressure, urine volume and consciousness level were decreased, and cyanosis, metabolic acidosis and hyperkalemia were subsequently emerged. She died on the 5th day of admission (2 weeks after the onset).

**Fig. 2:**
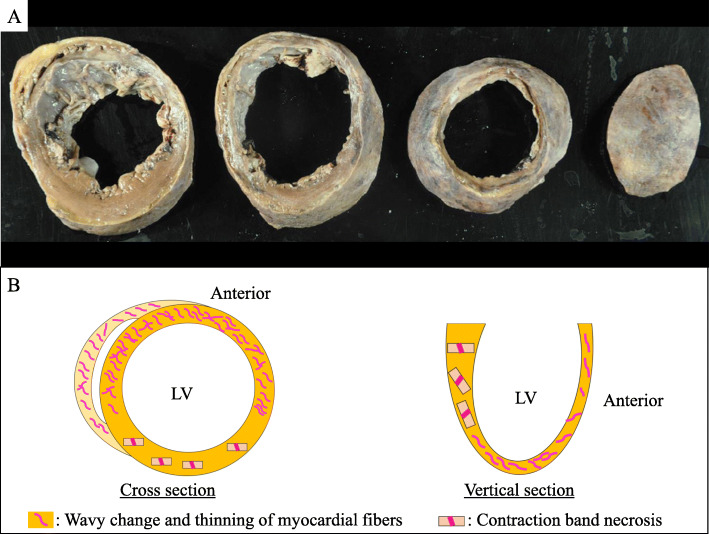
Cross sections and a schematic illustration of the heart. **a** The left ventricle was dilated at the basal to medial segment, and the medial to apical ventricular wall was thin and dull gray. **b** Remarkably, wavy change and thinning of the myocardium were seen on a broad area of the ventricle. On the other hand, contraction band necrosis was focally observed

The patient was 137 cm tall, with a body weight of 34 kg; her BMI was 18. At autopsy, the heart weighed 360 g and had a takotsubo-like shape (Fig. 1b, c). The epicardium had a reddish color and rough surface. There were no findings of thrombus, embolism, obstruction or severe stenosis of the coronary arteries (Fig. 1b-d). There was no cardiac rupture. Remarkably, the left ventricle was dilated in the basal to middle segment, and the ventricular wall was thin, especially at the middle to apical segment (Fig. 2a). Serous pleural effusion (left 400 mL, right 600 mL) was present. The lower lobes of bilateral lung were collapsed (left 250 g, right 270 g). Bleeding of the intestinal mucosa and moderate atherosclerosis were seen. The liver, left kidney, right kidney and spleen weighed 560 g, 60 g, 70 g and 25 g respectively. Microscopically, the heart showed notable degeneration and necrosis. Wavy change and thinning of the myocardium were diffusely observed especially on the apex and anterior to lateral wall of the left ventricle, interventricular septum and right ventricle, intermingled with interstitial fibrosis, hemorrhage and neutrophil infiltration (Fig. 2b, 3a-c). Contraction band necrosis was mainly observed on the posterior to inferior wall of the left ventricle (Fig. 2b, 3d). The liver showed centrilobular necrosis. Ischemic change was seen on the intestinal mucosa, suggesting ischemic mucosal hemorrhage.

**Fig. 3:**
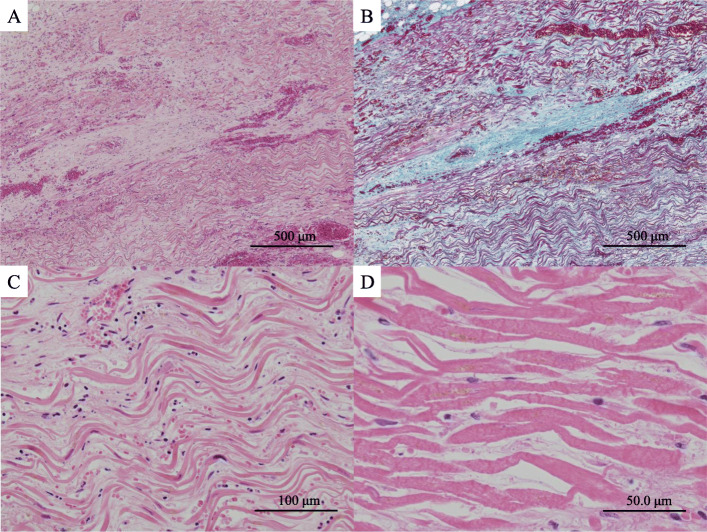
Microscopic view of the heart. **a**, **b** A low-power view of the apex of the left ventricle showed degeneration of the myocardium intermingled with interstitial fibrosis and hemorrhage. Bar = 500 μm (H&E staining; original magnification: × 40) (**a**). Bar = 500 μm (Masson trichrome staining; original magnification: × 40) (**b**). **c** A high-power view of the myocardium revealed remarkable degeneration and necrosis presenting wavy change and thinning. Bar = 100 μm (H&E staining; original magnification: × 200). **d** Contraction band necrosis was seen on the posterior to inferior wall. Bar = 50.0 μm (H&E staining; original magnification: × 400)

## Discussion and conclusion

The patient had several episodes of transient cardiac dysfunction within approximately 1 week before the hospitalization and echocardiography showed takotsubo-like shape on the admission. From epidemiological and clinical viewpoints, the symptoms of the patient in this case were consistent with takotsubo cardiomyopathy [1–3], and several myocardial ischemic episodes within 2 weeks before she died were thought to have been takotsubo myocardiopathy. This is the unique point of the present case. At autopsy, macroscopically the ventricle showed broad degeneration. Microscopy revealed remarkable wavy change and thinning of the myocardium with interstitial fibrosis on most of the ventricular wall and contraction band necrosis was focally recognized. Contraction band necrosis, which reflects a recent myocardial infarction that was partially reperfused, is a common microscopic finding of takotsubo cardiomyopathy, which is reversible cardiac dysfunction. On the other hand, wavy change and thinning or fibrosis are considered to reflect irreversible myocardial damage. Ischemic heart disease was one of differential diagnoses; however, there were no obstruction or severe stenosis of the coronary arteries, and clinical findings were compatible with takotsubo cardiomyopathy. Although the reason was not clear, broad myocardial irreversible or ischemic change might lead to hemorrhagic pericardial effusion or reddish color and rough changes of the epicardium.

Interestingly, in our case, macroscopically and microscopically diffuse irreversible myocardial damage was observed after the recurrence of takotsubo cardiomyopathy episodes within a short period of time. Further investigation is need; however, notably, this report suggests that the frequent recurrence of takotsubo cardiomyopathy causes irreversible severe myocardial damage and is life threatening.

## Data Availability

The dataset supporting the findings and conclusions of this case report is included within this article.

## Abbreviations

BNP: Brain natriuretic peptide
CK: Creatine kinase
CRP: C-reactive protein
BUN: Blood urea nitrogen
AST: Aspartate aminotransferase
ALT: Alanine aminotransferase
